# Lateral Geniculate Nucleus Volume Determined on MRI Correlates With Corresponding Ganglion Cell Layer Loss in Acquired Human Postgeniculate Lesions

**DOI:** 10.1167/iovs.63.9.18

**Published:** 2022-08-12

**Authors:** Cyril Fabian Simmen, Fabienne Catherine Fierz, Lars Michels, Njoud Aldusary, Klara Landau, Marco Piccirelli, Ghislaine Lieselotte Traber

**Affiliations:** 1 Department of Neurology, University Hospital Zurich, Clinical Neuroscience Center, University of Zurich, Zurich, Switzerland; 2 Department of Ophthalmology, University Hospital Zurich, University of Zurich, Zurich, Switzerland; 3 Department of Neuroradiology, University Hospital Zurich, Clinical Neuroscience Center, University of Zurich, Zurich, Switzerland; 4 Neuroscience Center Zurich, University of Zurich and Swiss Federal Institute of Technology Zurich, Zurich, Switzerland; 5 Department of Diagnostic Radiology, Faculty of Applied Medical Science, King Abdulaziz University, Jeddah, Saudi Arabia; 6 Department of Ophthalmology, University Hospital Basel, University of Basel, Basel, Switzerland; 7 Institute of Molecular and Clinical Ophthalmology Basel, Basel, Switzerland

**Keywords:** lateral geniculate nucleus, postgeniculate lesions, ganglion cell layer, optical coherence tomography, optic nerve

## Abstract

**Purpose:**

To quantitatively assess lateral geniculate nucleus (LGN) volume loss in the presence of lesions in the postgeniculate pathway and its correlation with optical coherence tomography retinal parameters.

**Methods:**

This was a case control study of patients recruited at the University Hospital Zurich, Switzerland. Nine patients who were suffering from lesions in the postgeniculate pathway acquired at least 3 months earlier participated. Retinal parameters were analyzed using spectral domain optical coherence tomography and a newly developed magnetic resonance imaging protocol with improved contrast to noise ratio was applied to measure LGN volume.

**Results:**

The affected LGN volume in the patients (mean volume 73.89 ± 39.08 mm^3^) was significantly smaller compared with the contralateral unaffected LGN (mean volume 131.43 ± 12.75 mm^3^), as well as compared with healthy controls (mean volume 107 ± 24.4 mm^3^). Additionally, the ganglion cell layer thickness corresponding with the affected versus unaffected side within the patient group differed significantly (mean thickness 40.5 ± 4.11 µm vs 45.7 ± 4.79 µm) compared with other retinal parameters. A significant linear correlation could also be shown between relative LGN volume loss and ganglion cell layer thickness decrease.

**Conclusions:**

Corresponding LGN volume reduction could be shown in patients with postgeniculate lesions using a newly developed magnetic resonance imaging protocol. LGN volume decrease correlated with ganglion cell layer thickness reduction as a sign of trans-synaptic retrograde neuronal degeneration.

The lateral geniculate nucleus (LGN) located in the thalamus is a relay center for the visual pathway, receiving information from the retinal ganglion cells and projecting axons via the optic radiations to the primary visual cortex (V1) in the occipital lobe of the brain.^1^^,^^2^

The functional integrity of the LGN can be examined by psychophysical evaluation of visual function, using perimetry. Common structural magnetic resonance imaging (MRI) until very recently lacked the resolution and accuracy needed for exact LGN visualization and volume quantification. Overcoming these limitations, a recently proposed MRI examination protocol now allows volumetric analysis of the LGN despite its small size.^3^

LGN volume may be altered by various pathologies affecting the visual pathway from the eye to the primary visual cortex. Atrophy of the LGN can result either from direct damage or from trans-synaptic degeneration. The latter can be caused either via anterograde (Wallerian) degeneration from the optic nerve axons,^4^ or from trans-synaptic retrograde neuronal degeneration (TRND) of neurons downstream from the LGN in the optic radiations or the primary visual cortex (V1) as demonstrated by Uggetti et al.^5^ in 1997. TRND has initially been observed in monkeys analyzing LGN volume and retinal damage in the ganglion cell layer (GCL) by conducting histological experiments.^6^^–^^9^ Human trials investigating this process have mostly focused on the relationship between occipital lesions and retinal nerve fiber layer (RNFL) as well as GCL measured by optical coherence tomography (OCT).^10^^–^^14^ TRND was first studied in congenital cases^5^ and subsequently in acquired pathologies as well.^14^^–^^17^

In MRI studies, optic tract degeneration was demonstrated as a result of occipital lobe lesions.^7^^,^^18^ As reported by Bridge et al.,^18^ there also seems to be qualitative evidence of LGN atrophy ipsilateral to occipital lobe lesions on T1-weighted MRI. More recently, LGN volume has been successfully measured by MRI in patients with Leber's hereditary optic neuropathy, demonstrating the effects of anterograde degeneration on LGN volume.^19^ However, based on our review of the literature using the Web of Science and PubMed databases, we believe that no study has yet examined the relationship between acquired lesions in the postgeniculate pathway and LGN volume using MRI. The main purpose of the current study was to quantitatively assess LGN volume loss in the presence of lesions in the postgeniculate pathway, using the dedicated and optimized imaging protocol proposed by our group.^3^ Further, we assessed the correlation of LGN volume loss with changes in retinal OCT parameters, hypothesizing that retinal layers would also be affected as a consequence of TRND.

## Methods

### Study Design, Study Population, and Inclusion and Exclusion Criteria

This case control study was conducted at the University Hospital Zurich, Switzerland. Patients with acquired diseases affecting the postgeniculate visual pathway were consecutively recruited meeting the following inclusion criteria: aged 18 years or older, lesions of the occipital lobe or optic radiations (not involving the LGN itself) present for at least 3 months before recruitment, homonymous visual field defects, and normal intraocular pressure (IOP) in both eyes. We decided on 3 months because it can be reasonably hypothesized that TRND may take some time to result in a measurable change in volume in the LGN. After 3 months, most of the decrease could be expected to have occurred as described by Mihailović et al.^20^ in 1971 in animals and by Jindahra et al. in 2009^14^ and Bridge et al. in 2011^18^ in humans. Patients with intraocular pathologies that would adversely affect visual acuity, visual field, or retinal anatomy were excluded and no patient had any neurological lesions other than those in the optic radiations or the visual cortex. We also excluded patients with a history of ocular or cerebral trauma or surgery (except for uncomplicated cataract surgery). A total of nine patients were recruited; two patients had bilateral lesions.

The control group (*n* = 9) consisted of age- and gender-matched healthy volunteers. The inclusion criteria for the control group were best-corrected visual acuity of 20/20 and no history of previous ophthalmologic or neurologic disease or intervention except for previous cataract surgery.

### General Assessment

All subjects underwent ophthalmologic examinations including best-corrected visual acuity, static or kinetic perimetry, IOP measurements, slit-lamp examination, and fundoscopy.

### Spectral Domain OCT Imaging

We obtained OCT images with automated segmentation using the Heidelberg Spectralis Spectral Domain OCT device (Heidelberg Eye Explorer Version 1.9.10, Viewing Module 6.0.14, Acquisition Module 6.0.13; Heidelberg Engineering, Heidelberg, Germany). The peripapillary ring scan and the posterior pole scan were used for analysis with 61 consecutive vertical B-scans (scan angle, 30°; 121 µm between B-scans, automatic real time function 100) in high-resolution mode. We measured the peripapillary RNFL thickness as well as the macular retinal thickness (RT) and GCL thickness in all patients and healthy controls using the automatic segmentation provided by the manufacturer. Manual correction was performed in case of segmentation algorithm failure. Taking into account the anatomy of the anterior visual pathway with decussation of the axons of nasal retinal ganglion cells in the chiasm, these measurements were further subdivided into nasal and temporal anatomical sectors for further correlation with the LGN structure. The posterior pole scan was divided into 64 concentric squares. Of the central 16 squares, the nasal and temporal squares respectively were used for statistical analysis. Accordingly, the nasal and temporal sectors of the peripapillary RNFL ring scan were analyzed.

### MRI Acquisition and Analysis

LGN volume was derived from Magnetization Prepared Rapid Gradient Echo images using isotropic acquisition resolution (0.75 mm) with a relatively short running time of approximately 15 minutes. We used a newly developed protocol for LGN volume quantification with an improved contrast to noise ratio by subtracting grey matter nulled images from white matter nulled images. The detailed imaging protocol and the rationales of the MRI protocol optimization has been outlined in detail by Aldusary et al.^3^ Using this method, the imaging standard deviation of the volume quantification is approximately 2%, which is much smaller than the 10% standard deviation of LGN volumes in healthy populations. The use of several raters further improves the quantification, see Aldusary et al. 2018 supplementary material for further details.

For this study, three independent raters who had been trained by a neuroradiologist used the program MIPAV (NIH, Center for Information Technology; Bethesda, MD) to manually segment the LGN structures on all images where they were clearly visible and could be highlighted against the rest of the thalamus. The inter-rater reliability using this method has been shown to be quite high.^3^ The average of the three raters’ measurements was used in the analysis.

### Statistical Analysis

In a first analysis, we investigated unilaterally affected patients comparing affected (*n* = 7) and unaffected (*n* = 7) sides. The two patients with bilateral lesions were excluded from intraindividual comparison of LGN size in the patient group. In a second analysis, we compared all the affected sides of the patient group (*n* = 11) and the control group with the corresponding sides (*n* = 11). The unpaired *t*-test was used to compare values between continuous variables. The Mann–Whitney *U* test was used for categorical variables. We conducted all tests as two-sided. A *P* value of 0.05 or less was considered statistically significant. Statistical analyses were calculated using the software R, version 3.5.0 (The R Foundation for Statistical Computing, Vienna, Austria) and MATLAB (Matlab, The MathWorks Inc., Natick, MA).

## Results

Nine patients (3/9 males; mean age, 57.4 ± 17.32 years) and nine healthy controls (3/9 males; mean age, 52.1 ± 18.4 years) were recruited. There was no statistical difference in age and gender between the two groups (Table 1).

**Table 1.** Baseline Characteristics of the Patient and Control Groups

Variable	Controls	Patients	*P* Value
*N*	9	9	
Unilaterally affected	NA	7	
Bilaterally affected	NA	2	
Age	52.1 ± 18.4	57.4 ± 17.3	0.54
Sex			
Male	3 (33.3)	3 (33.3)	
Female	6 (66.7)	6 (66.7)	
Diagnoses			
Neoplastic	NA	2 (22.2)	
Vascular	NA	7 (77.8)	

NA, not applicable.

Values are number (%) or mean ± standard deviation.

Table 2 summarizes the individual demographic and clinical characteristics of the patients, including diagnosis and a description of the visual field defect. Four subjects had an ischemic insult, three had a cerebral hemorrhage, and two had a neoplastic disease. In seven patients the lesion affected the primary visual cortex (V1) and in three patients the optic radiations were damaged. Of these, two patients had lesions on both hemispheres of the brain.

**Figure 1.**
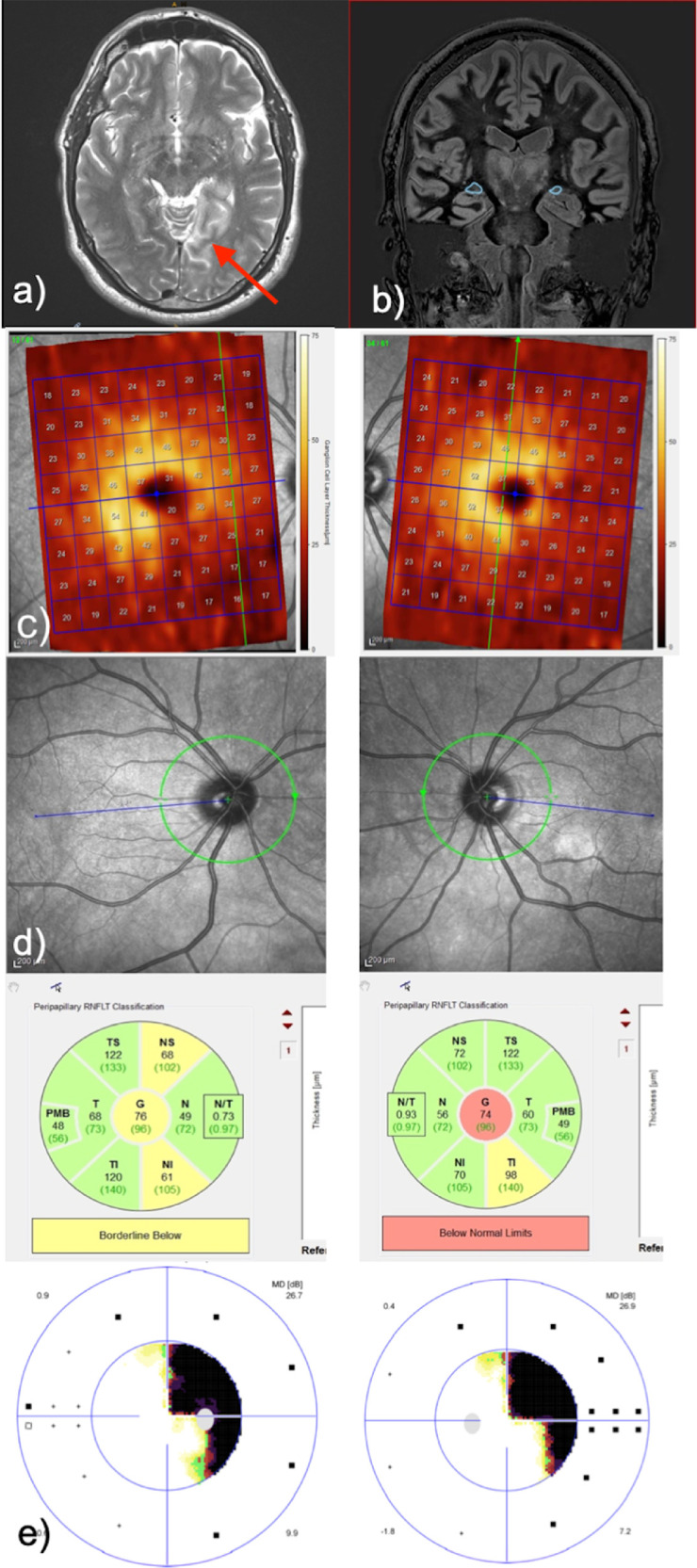
Patient 5 with ischemic insult on the left side, as indicated by arrow. (**a**) Axial T2-weighted MRI sequence showing the lesion. (**b**) Sequence for LGN segmentation with highlighted LGNs. Note the marked asymmetry in the size of the right and left LGNs. (**c**) Heat map of GCL thickness measurements by OCT (posterior pole scan). (**d**) Peripapillary RNFL thickness measurements by OCT. (**e**) Homonymous visual field defects to the right demonstrated by automated static perimetry.

Figure 1 presents clinical and imaging findings of patient 5 with a diagnosis of ischemic insult.

**Figure 2.**
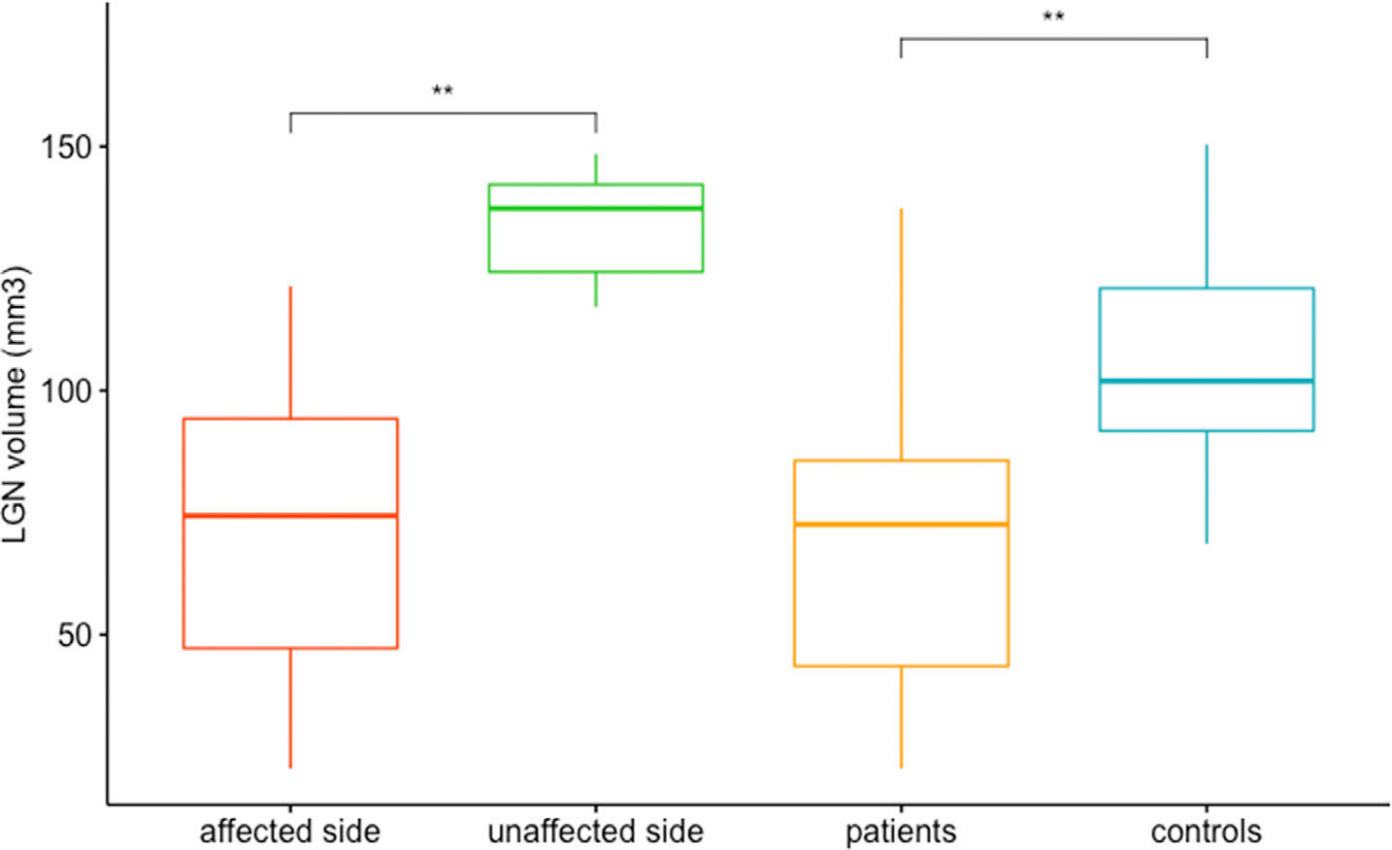
LGN volume comparison between affected and unaffected sides within the unilaterally affected patient group (*P* = 0.0071), and between affected LGN volume of the patients and corresponding side in the matched LGNs in the healthy control group. *P* = 0.0063. **Significant result.

First, we compared affected (*n* = 7) and unaffected (*n* = 7) sides in patients with unilateral lesions (Fig. 2). The LGN volume on the affected side (mean volume, 73.89 mm^3^ ± 39.08) was smaller compared with the unaffected side (mean volume, 131.43 mm^3^ ± 12.75), which reached statistical significance (*P* = 0.007136).

When comparing all affected LGN sides (*n* = 11) with their matched controls (*n* = 11) the affected LGN volume in the patient group (mean volume, 68.6 ± 32.8 mm^3^) was significantly smaller (*P* = 0.00627) compared with the corresponding side in the control group (mean volume, 107.0 ± 24.4 mm^3^) (Fig. 2).

For RNFL and RT measurements, no difference was found neither between affected and unaffected side (*P* = 0.71493 and *P* = 0.29733) nor between affected side and matched side in controls (*P* = 0.66647 and *P* = 0.48943).

**Figure 3.**
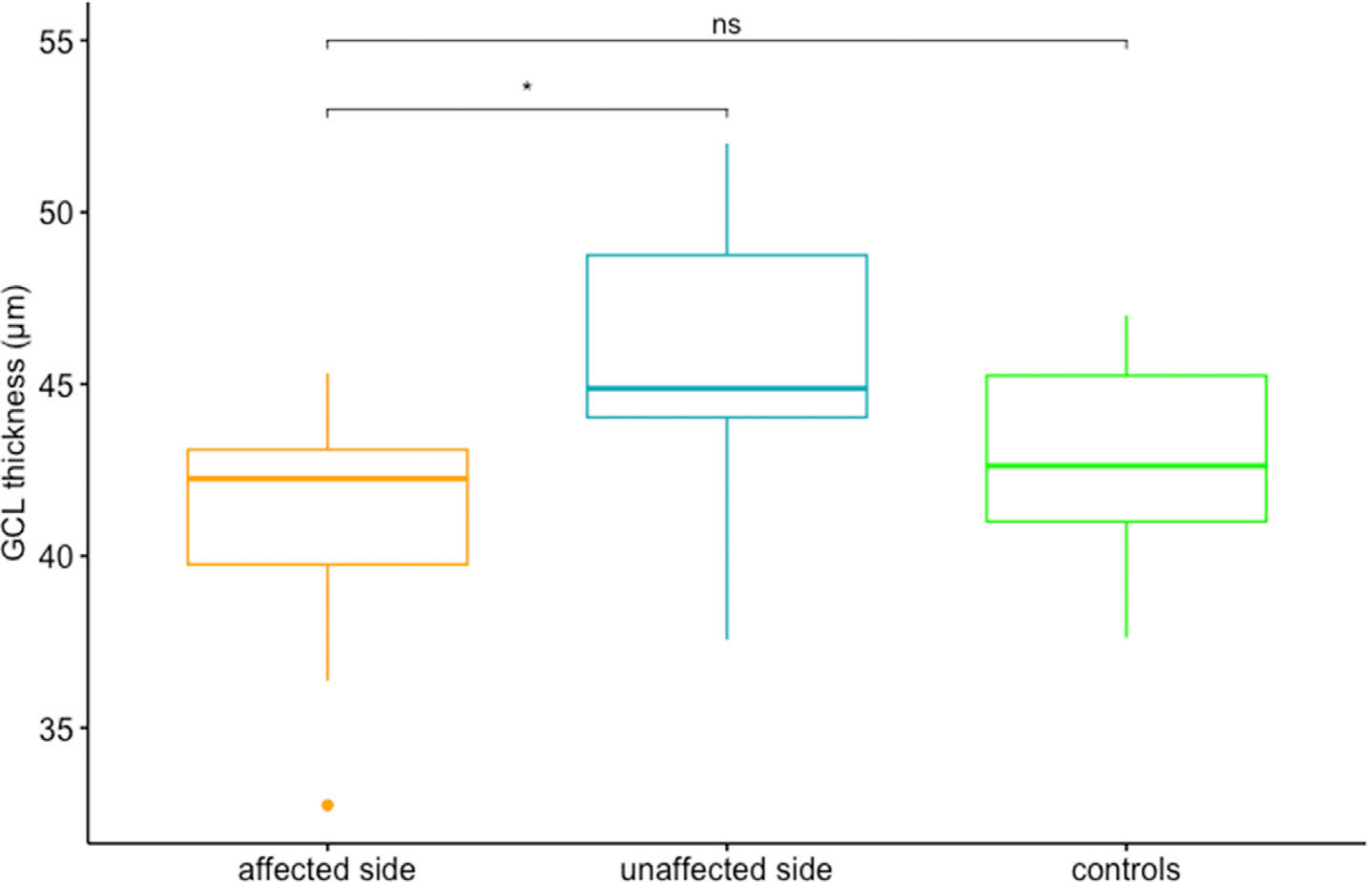
Comparison of GCL thickness corresponding to affected versus unaffected side within patient group (*P* = 0.04) and to corresponding retina in healthy controls (*P* = 0.46). *Significant result; ns, nonsignificant result.

Regarding other OCT parameters, GCL thickness corresponding to the affected side (mean thickness, 40.5 ± 4.11 µm) versus unaffected side (mean thickness, 45.7 ± 4.79 µm) within the unilaterally affected patient group (*n* = 7) showed a significant difference (*P* = 0.04) (Fig. 3). However, the difference in GCL thickness between the affected side in the patient group (*n* = 11; mean thickness, 40.9 ± 3.66 µm) and healthy controls (*n* = 11; mean thickness, 42.1 ± 3.44 µm) was only observed as a trend without reaching statistical significance (*P* = 0.46) (Fig. 3).

**Figure 4.**
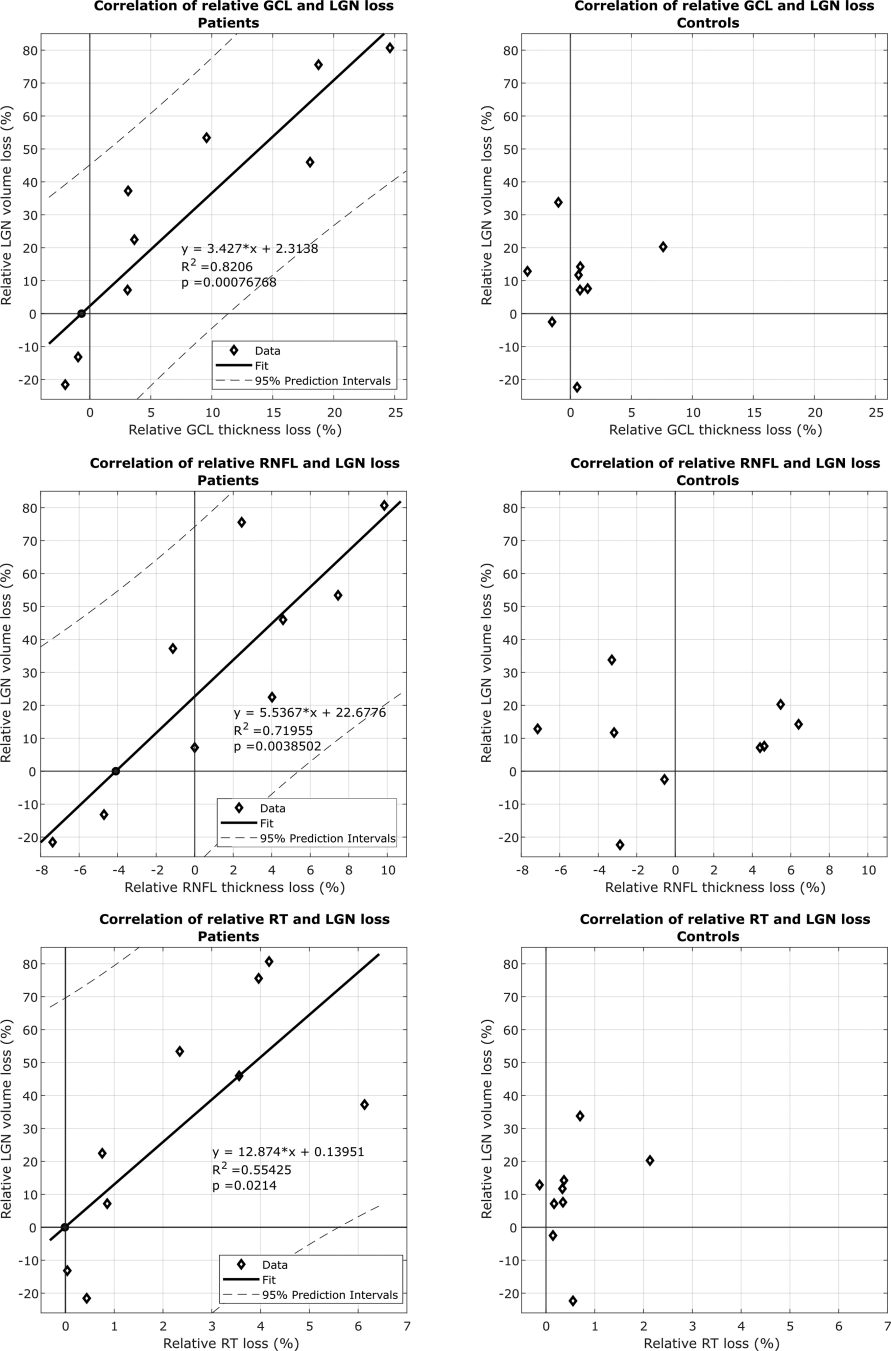
(**a**) Correlation of relative GCL thickness and LGN volume loss in patients and healthy controls. (**b**) Correlation of relative RNFL thickness and LGN volume loss in patients and healthy controls. (**c**) Correlation of relative RT and LGN volume loss in patients and healthy controls.

We then correlated the relative (to the contralateral side) loss of GCL thickness and relative LGN volume loss in patients, which proved to be a significant linear correlation (*P* = 0.000763) (Fig. 4). Accordingly, a weaker correlation was found for RNFL (R^2^ = 0.720; *P* = 0.0039) and the RT measurements (R^2^ = 0.554; *P* = 0.0214). For the healthy controls, no such relationship could be established.

## Discussion

The aim of our study was to examine the effect of acquired postgeniculate lesions on LGN volume using a newly developed MRI protocol.^3^ Our study results confirm that state-of-the-art imaging techniques show ipsilateral LGN volume loss in patients with acquired postgeniculate lesions in vivo, which had only been shown in histological animal studies so far.^8^^,^^9^

In addition, we found further evidence of TRND using OCT, demonstrating GCL thickness reduction following postgeniculate lesions in our patients and its correlation with the extent of LGN volume loss. Given the lack in precision of LGN imaging, research in human studies has until recently mostly focused on detecting TRND of postgeniculate lesions by means of retinal OCT imaging.^10^^,^^13^

Optic tract atrophy in the context of TRND has also been studied by MRI, but so far without volume quantification of LGN volume as used in our study.^15^^,^^18^ However, recent advances in MRI techniques towards visualizing small anatomical brain structures have made this a field of increasing importance.^3^ LGN visualization has so far been limited to studies involving anterograde degeneration in ophthalmological diseases such as glaucoma or Leber's hereditary optic neuropathy.^19^^,^^21^ Our study shows a clear ipsilateral LGN volume decrease compared with the unaffected side in patients with a postgeniculate visual pathway lesion (*P* = 0.007). The median affected LGN volume is also significantly smaller when compared with the side matched LGN volume of the control group (*P* = 0.006). There was the same trend for a thinner GCL on the affected side on an intraindividual level (*P* = 0.04), but less so when comparing GCL thickness corresponding with the ipsilesional side in patients with the matched side in controls (*P* = 0.46). Although the RNFL thickness showed a weaker correlation with the LGN volume, RT measurements could not detect any asymmetry. This indicates that GCL measurements are more sensitive to reveal damage of the visual pathway compared with the peripapillary RNFL. This idea is also strongly supported by our finding of a highly linear correlation between relative GCL thickness and LGN volume loss in our patients, which was better than the correlation with either RNFL thickness or RT. As expected, the same analyses in the control group did not show any correlation. The GCL's greater sensitivity compared with RNFL thickness has been described by different authors for various ophthalmologic pathologies.^13^^,^^22^^–^^25^ This finding is highly relevant for the clinical evaluation of patients with afferent neuro-ophthalmic disorders, because GCL loss may be the earliest sign of neuronal damage when the RNFL may yet seem normal.

Owing to the topographical organization of the visual pathway, a correlation between LGN volume loss and extent of retinal layer loss is expected in the presence of TRND. Indeed, we found a strong correlation for relative GCL loss and LGN volume loss. A weaker correlation was found for RNFL and RT decrease (Fig. 4). A negative relative LGN loss was found in two patients, one of whom had bilateral lesions in the postgeniculate pathway with a relative asymmetry of 22% for LGN volume and 2% for GCL asymmetry. The other presented with a transient homonymous visual field defect secondary to a cerebral hemorrhage affecting V2/V3 (Table 2, patient 4), that presumably did not result in neuronal damage sufficient to result in a correlation. Another explanation in the latter patient would be the fact that only V2 and V3 were affected, therefore, potentially not giving raise to retrograde axonal degeneration reaching the LGN. However, it has been argued that lesions of V2/V3 may in fact directly damage optic tract fibers.^26^

**Table 2.** Demographic and Clinical Patient Characteristics

Case	Age (y)^*^	Sex	Diagnosis	Side	Exact Lesion Location	Visual Field Defect	Age of Lesion (mo)^†^
1	85	F	Ischemic insult	Right/left	V1 right and OR left	Homonymous left superior quadrantanopia	13
2	73	F	Brain metastasis (peritoneal carcinoma)	Right/left	V1 right and OR left	Incomplete homonymous left hemianopia (central)	14
3	72	F	Cerebral hemorrhage	Left	V1–V2	Homonymous central right inferior defect	63
4	64	F	Cerebral hemorrhage	Right	V2–V3	Homonymous incomplete left hemianopia (inferior>superior)	3
5	55	M	Ischemic insult	Left	V1	Homonymous incomplete right hemianopia (superior>inferior)	18
6	52	M	Ischemic insult	Right	V1–V2	Homonymous central left inferior defect	7
7	44	M	Cavernoma	Left	V1	Incomplete homonymous right superior quadrantanopia	4
8	38	F	Cerebral hemorrhage	Left	OR	Homonymous right hemianopia	10
9	34	F	Ischemic insult	Left	V1	Homonymous incomplete right superior quadrantanopia	8

OR, optic radiations; V1, primary visual cortex; V2, secondary visual cortex; V3, tertiary visual cortex.

* At the time of lesion occurrence.

† At the time of first examination.

A quantitative volumetric assessment of the LGN using our dedicated MRI protocol will enable future longitudinal studies to gain insight into the dynamics and extent of LGN atrophy in relation to temporal lesion onset. Progressive thinning of the RNFL in the context of occipital lobe/optic radiation damage owing to stroke was first reported by Jindahra et al. in 2011.^12^ Likewise, a progressive decrease in the GCL thickness over time was observed in acquired homonymous hemianopia.^27^ With refined MR and OCT in vivo imaging techniques at hand, the process of retrograde trans-synaptic degeneration can now potentially be studied over the whole course of the visual pathway in humans.

As a limitation, the present study only provides evidence for LGN volume loss correlation with GCL loss correlation in a relatively small patient population and should be reproduced in a larger population. Correlating the findings with the time from lesion onset and extent of visual field deficits would enhance the clinical impact of the findings. However, standardized automated visual field data were not available at different time points in our study.

As a technical limitation, the smaller size of the LGN in pathological cases make the precise volumetric assessment even more difficult. Another source of noise are the physiological LGN volume variations between humans as seen in the control group in Figure 2, which had been described previously by Aldusary et al.^3^ as well as the physiological relative side variation between the two LGNs as seen in Figure 4.

## Conclusions

Using a newly developed MRI protocol in a clinical setting, we were able to demonstrate ipsilateral LGN volume reduction in patients with postgeniculate lesions. Moreover, the LGN volume decrease correlation with GCL thickness reduction in these patients demonstrated human in vivo trans-synaptic retrograde degeneration.
